# Progress in Medicine

**Published:** 1897-02-20

**Authors:** 


### PROGRESS IN MEDICINE.

### DISEASES OF CHILDREN.

**Diseases of Infants during Lactation.**—In a clinical lecture, Dr. Marfan[1] discusses abdominal intumescence and its causes in breast-fed babies. Omitting the rare cases due to solid or fluid swelling, he states that there are two varieties of enlarged abdomen—namely, the tympanitic and the flabby. The characteristics of the tympanitic abdomen are tension, hardness, and resonance on percussion, and the condition is usually ascribed to an abnormal production of gas in the intestine, with consequent distension and paresis of the intestinal walls. On the other hand, Dr. Marfan believes that congestive turgescence—intestinal and hepatic—is a more important factor in producing the distension than fermentative changes in the contents of the bowel. The tympanites is usually associated with diarrhœa, and both are frequently acute and transitory, but in persistent cases a condition of entero-colitis is present, due to ulceration in the lymph follicles of a purely irritative and non-specific character. The enlarged flabby abdomen is soft, limp, and projecting at the sides like the belly of a frog. Umbilical and inguinal herniæ are common in this condition, and there may be prolapse of the bowel between the recti muscles, or even prolapse of the rectum, as pointed out by Dr. Comby. The usual history in such cases is that, after a period of attacks of diarrhœa alternating with constipation and tympanitic abdomen, the patient becomes cachectic and wasted, and the abdomen large and flabby. Such cases have usually been classed as examples of tabes mesenterica; but this view is not supported by examination after death, and Dr. Marfan has found one marked pathological change—namely, elongation of the intestine as a whole, a condition to which he gives the name of dolichoenterica. Under normal circumstances the length of the intestine in a child is from seven to eight times the height of the body, and in cases of flabby abdomen it was found to be nine to twelve times. The elongation affects both the small and the large intestine, but is comparatively more marked in the former. The condition is a very persistent one, and Dr. Marfan suggests that it may be the cause of enteroptosis and splanchnoptosis in adult life.

In a paper on "The Indigestion of Breast Babies"

Dr. James Carmichael[2] sounds a note of warning as to the effect that the present system of over-educating girls has in unfitting them from properly discharging their maternal duties in later life. Many women are most anxious to nurse their children, but are unable to do so from causes which are distinctly preventable. The indigestion of breast-fed babies is more often intestinal than gastric, and diarrhœa is consequently more marked than vomiting. The infant becomes soft and flabby, is irritable, crying, and unsatisfied, the skin becomes harsh and dry, and the tongue is red and furred. The motions are loose, pale, grey, or green in colour, and may contain mucus and blood. In such cases one must carefully examine into the state of the mother's health, and see whether she suffers from any disease, functional or organic, which incapacitates her from nursing. The most common cause of the indigestion is too frequent and irregular suckling, which induces an over-concentrated condition of the milk. In other cases the diet and habits of the mother must be revised, plenty of good plain food ordered, and a due amount of out-of-door exercise. If, on chemical examination of the milk, there is found a deficiency of solid materials, then more frequent suckling is to be ordered; if an excess of solids, then less frequent suckling, more exercise, and a larger amount of fluid nutriment are indicated. The fatty materials in the milk will vary with the quantity of animal food taken by the mother. The proteid constituents are to be regulated by exercise in the open air, active exercise diminishing the amount of proteids.

**Congenital Tumour of the Face.**—An extremely interesting and probably unique case of this nature is related by Mr. J. Rutherford Morison.[3] The patient when first seen was eleven months old, but the tumour had existed from birth, and had not increased out of proportion to the child's growth. Bleeding from the tumour occurred at the age of two months, and on subsequent occasions, a considerable quantity of blood being lost. When the child began to cut his teeth at eight months two teeth also appeared on the surface of the tumour. At first sight the growth looked like a large orbital sarcoma concealing the eye. The mass appeared as if coming out of an opening in the side of the nose, and carrying the skin of the nose forward in its upper part, the lower part being covered with apparently normal mucous membrane. A sulcus about half an inch deep and ending blindly surrounded the tumour. At the outer edge of this sulcus there were two tubercles, looking like two maxillary processes with a harelip between. One of the tubercles had cut a well-developed temporary incisor tooth, and the other was about to do so. The nostrils, mouth, and palate were normal, and there was no communication between these passages and the tumour. Mr. Morison removed the growth with a gouge after dissecting a flap of skin off the upper surface. The base of the tumour was found to be solid, and entirely blocked the upper part of the nose. The child made a good recovery. Mr. J. H. Targett has examined the growth and regards it as a teratoma, the imperfect lip and the two tooth germs being sufficient evidence of fœtal elements. Histologically there was little more to be seen than fibrous and mucoid tissues irregularly mingled. Dr. J. W. Ballantyne, in discussing the case from a teratological standpoint, remarks that "the mass may be regarded as a teratoma, as an exoprosopus amorphus, or as an accessory upper jaw."

**Tuberculosis of the Intestine by Ingestion.**—Marfan and Apert[4] report a case of this probably not very uncommon condition. A child died at the age of sixteen months, primarily from diarrhœa, and the autopsy revealed an enormous mass of tuberculous mesenteric glands, with tuberculous ulceration of the large and small intestine. There were a few small greyish granulations in the lungs, with two foci of broncho-pneumonia at the right base, but no evidence of tuberculous disease in the liver, spleen, heart, or kidneys. The patient had been entirely breast-fed, with the exception of certain occasions on which, owing to the mother's absence at work, cow's milk was given. At the age of nine months a suppurative adenitis in the neck had developed, and it was noticed that the gums were ulcerated and unhealthy. Diarrhœa came on at the age of thirteen months, and persisted until death. There was a history of tuberculous disease in the family. The authors are of opinion that the first focus of disease was the mouth and the glands in the neck, and that later on the intestine was infected. The source of the infection may have been the milk of the mother, or of the cow, or through the medium of soiled substances put in the mouth. The occurrence of tuberculosis of the intestine is exceptional in the first two years of life, and it may be acquired in various ways: (1) As part of an acute miliary tuberculosis, or (2) from the swallowing of tuberculous sputum in chronic pulmonary tuberculosis, or (3) from the ingestion of tuberculous food, and especially of milk from a cow affected with that disease.

**Nervous Symptoms in Rickets.**—Dr. A. Jacobi[5] points out how important it is that the earliest signs of rickets should be recognised, and the disease at once treated. Amongst these he lays stress on sweating at night, especially that about the head, which leads to restlessness and rubbing of the head on the pillow until the occipital region becomes entirely denuded of hair. Hydrocephalus is common, and the prognosis is better in rickets than in other affections. Laryngismus stridulus is in the great majority of cases dependent on cranial rachitis. With this is frequently combined a tendency to attacks of tetany—also a symptom or associate of rickets. He discusses the facial symptom of tetany—the so-called Chvostek's sign—which is the production of a spasm of the upper eyelid and ala nasi by pressing on or tapping the skin over the zygomatic arch. He has found this condition present in many cases of tetany, but it is not absolutely diagnostic, as it may in some cases be absent, and it also occurs in connection with other affections, for example, in epilepsy, chlorosis, and neurasthenia.

Dr. John Abercrombie[6] also writes on the facial irritability in tetany and laryngismus stridulus. He states that, "under certain circumstances, on roughly passing the back of the nail over the skin just in front of the parotid region, the orbicularis palpebrarum, the levator alæ nasi, and the levator anguli oris will be seen to twitch smartly, just as if an electric current had been passed through them." This twitching is generally more marked on one side than on the other. The other accompanying conditions he has noted are an exaggerated activity of the patellar reflexes, and a general myotatic irritability. He has never failed to elicit this phenomenon of facial irritability in cases of tetany or laryngismus stridulus, but it also occurs apart from these disorders, for example, in anæmia, although the evidence is strong that in all cases rickets is the basis on which the muscular or nervous irritability develops.

**Cyclic Albuminuria in a Family.**—While examining the urine of a chlorotic girl of fifteen years, Schön[7] found albumen to be present, and on repeated testing this albuminuria proved to be of the cyclic variety. The albumen appeared about eleven a.m., reached its maximum amount about three p.m., and then gradually diminished until it had completely disappeared by eight p.m. The urine of two sisters and two brothers of the patient was examined, and a similar condition of albuminuria was found to exist in one of the sisters, a girl of thirteen, who was also chlorotic. Treatment of the chlorosis had no effect on the albuminuria.

**Cirrhosis of the Liver.**—In a clinical lecture on this subject, Dr. H. D. Rolleston,[8] after touching on the peculiar form of cirrhosis due to congenital syphilis, and the fibrotic changes of the liver met with in rickets, discusses those varieties of cirrhosis which in children are comparable to a similar condition in adults. The liver may be either larger or smaller than normal, the former being described as hypertrophic cirrhosis with chronic jaundice. Amongst the causes he enumerates alcohol, or vinegar, over-stimulating diet, the infectious fevers, *e.g.*, measles or scarlet fever, and some forms of anæmia, *e.g.*, splenic. In all cases there is probably some poison acting on the liver, originating possibly in the contents of the intestine, or produced in the system and conveyed to the liver by the portal vein, the hepatic artery, or the bile ducts. Clinically the disease is characterised by emaciation, dry skin, ascites, jaundice, diarrhœa, and hæmorrhages. Biliary toxæmia may lead to headache and vomiting. In rare instances the symptoms are entirely nervous, and cirrhosis, though practically the only lesion found post-mortem, is never suspected during life. These symptoms are progressive paralysis, loss of mental power going on to idiocy, fever, and marked prostration. The diagnosis, in the nervous form, presents great difficulty, but in the ordinary type of the disease, with jaundice, ascites, and a big liver, it is comparatively easy. If ascites alone is present, the disease is apt to be mistaken for tuberculous peritonitis, and in such a case the absence of enlarged mesenteric glands, the tense not doughy feel of the abdomen, and the occurrence of hæmorrhage are in favour of cirrhosis. The prognosis is extremely bad, although in some cases it is possible that the disease may be arrested. In the treatment all stimulating foods and drinks must be carefully avoided, and milk and fatty foods ordered. Fresh air and exercise are beneficial, provided ascites is not present. This latter condition is usually a sign that death is not far distant, and inasmuch as paracentesis gives relief, it should not be deferred after the abdomen has become much distended. As regards drugs, mercury and the iodide of iron are probably the most serviceable, and the bowels may be regulated with Carlsbad salts or some other saline aperient.

[1] The Medical Week, March 13, 1896. [2] Edin. Med. Jour., Aug., 1896. [3] Edin. Med. Jour., Aug., 1896. [4] Rev. Mens. des Malad. de l'Enfance, June, 1896; and Am. Jour. Med. Science, Aug., 1896. [5] Arch. of Pediat, Nov., 1896. [6] Ibid. [7] Jarhb. f. Kinderheil, 1896, Bd. XLI., S. 307. [8] Clin. Jour., Sept. 9, 1896.

